# Chronic post-COVID-19 syndrome and chronic fatigue syndrome: Is there a role for extracorporeal apheresis?

**DOI:** 10.1038/s41380-021-01148-4

**Published:** 2021-06-17

**Authors:** Stefan R. Bornstein, Karin Voit-Bak, Timo Donate, Roman N. Rodionov, Raul R. Gainetdinov, Sergey Tselmin, Waldemar Kanczkowski, Gregor M. Müller, Martin Achleitner, Jun Wang, Julio Licinio, Michael Bauer, Allan H. Young, Sandrine Thuret, Nicole Bechmann, Richard Straube

**Affiliations:** 1 Department of Medicine III, University Hospital Carl Gustav Carus, Technische Universität Dresden, Dresden, Germany; 2 Faculty of Life Sciences and Medicine, Division of Diabetes and Nutritional Sciences, King’s College London, London, UK; 3 Department of Endocrinology and Diabetology, University Hospital Zurich, Zurich, Switzerland; 4 Paul Langerhans Institute Dresden (PLID), Helmholtz Center Munich, University Hospital Carl Gustav Carus and Faculty of Medicine, TU Dresden, Dresden, Germany; 5 Zentrums für Apherese- und Hämofiltration am INUS Tagesklinikum, Cham, Germany; 6 Institute of Translational Biomedicine St. Petersburg State University, Saint Petersburg, Russia; 7 St. Petersburg State University Hospital, St. Petersburg State University, Saint Petersburg, Russia; 8 State University of New York, Upstate Medical University, Syracuse, NY USA; 9 Department of Psychiatry and Psychotherapy, Carl Gustav Carus University Hospital, Technische Universität Dresden, Dresden, Germany; 10 Centre for Affective Disorders, Department of Psychological Medicine, Institute of Psychiatry, Psychology & Neuroscience, King’s College London, London, UK; 11 Department of Basic and Clinical Neuroscience, Institute of Psychiatry, Psychology & Neuroscience, King’s College London, London, UK; 12 Institute of Clinical Chemistry and Laboratory Medicine, University Hospital Carl Gustav Carus, Medical Faculty Carl Gustav Carus, Technische Universität Dresden, Dresden, Germany; 13 Department of Experimental Diabetology, German Institute of Human Nutrition Potsdam-Rehbruecke, Nuthetal, Germany; 14 German Center for Diabetes Research (DZD), Munich-Neuherberg, Germany

**Keywords:** Neuroscience, Diseases, Stem cells

## Abstract

As millions of patients have been infected by SARS-CoV-2 virus a vast number of individuals complain about continuing breathlessness and fatigue even months after the onset of the disease. This overwhelming phenomenon has not been well defined and has been called “post-COVID syndrome” or “long-COVID” [1]. There are striking similarities to myalgic encephalomyelitis also called chronic fatigue syndrome linked to a viral and autoimmune pathogenesis. In both disorders neurotransmitter receptor antibodies against ß-adrenergic and muscarinic receptors may play a key role. We found similar elevation of these autoantibodies in both patient groups. Extracorporeal apheresis using a special filter seems to be effective in reducing these antibodies in a significant way clearly improving the debilitating symptoms of patients with chronic fatigue syndrome. Therefore, such a form of neuropheresis may provide a promising therapeutic option for patients with post-COVID-19 syndrome. This method will also be effective when other hitherto unknown antibodies and inflammatory mediators are involved.

## Introduction

Myalgic encephalomyelitis/chronic fatigue syndrome (ME/CFS) is an underestimated frequent and debilitating disorder with a major impact on quality of life. Underlying pathomechanisms involve both an initiation or trigger by viral disease and in a significant subset an autoimmune etiology. A hallmark of viruses linked to ME/CFS is the ability to establish persistent and chronic infections. Chronic low-level inflammation and activation of cell-mediated immunity with an increase in inflammatory mediators contribute to the clinical symptoms of this disease including fatigue, fever, sleep, and cognitive disorders [2]. Inflammation of glial cells correlated by inflammatory cytokines and neuronal stimulation may induce chronic pain [3]. Immune dysregulation in ME/CFS has been frequently observed involving not only changes in cytokine profiles, but also in immunoglobulin levels, T- and B-cell phenotype and a decrease in natural killer cell cytotoxicity. Recently, autoantibodies against neurotransmitter receptors have been shown to be elevated in patients with ME/CFS. This provides the opportunity to employ therapeutic strategies targeting the removal of these autoantibodies either by B-cell depletion or by apheresis. A chronic post-viral syndrome characterized by chronic fatigue, variable non-specific myalgia, depression, and sleep disturbances has previously been reported following SARS coronavirus infection [4]. In a pandemic with millions of people affected worldwide, this has now become a threatening and overwhelming problem for both our healthcare systems and economy for years to come. Therefore, there is urgent need to investigate the mechanisms of these devastating sequelae of COVID-19 and to search for effective treatment options.

## Role of neurotransmitter receptor antibodies in ME/CFS

A characteristic feature of ME/CFS is a dysregulation of the autonomic sympathetic and parasympathetic nervous system leading to the clinical symptoms of this disorder. This includes dysfunction of the vasomotor and gastrointestinal system and increased sensitivity to pain [5]. In this context, it is of great interest that autoantibodies against the M1 acetylcholine receptors (AChR) were demonstrated in these patients. Occurrence of these autoantibodies was associated with reduced binding of M1 AChR ligand in brain analyzed by PET imaging [6, 7]. Furthermore, autoantibodies against β1 and β2 adrenergic receptors (AdR) were found in patients with ME/CFS [8]. It has been previously shown that β-AdR autoantibodies occurring in patients with dilated cardiomyopathy orthostatic hypotension and postural orthostatic tachycardia syndrome contribute to the autonomic dysfunction and fatigue in these patients. These are also the features shared with patients suffering from ME/CFS. The exact mechanisms triggering the generation of these neurotransmitter receptors in CFS patients remain elusive. However, an induction by viral toxins is most likely.

Similarly, it is unclear how elevated antibody titers are correlating with disease symptoms. However, it is of great interest that there is a significant correlation of these neurotransmitter receptors with immunoglobulin levels, T-cell activation, and other autoantibodies such as ANA or TPO antibodies [8] suggesting a broad and complex activation of the immune system.

## Targeting neurotransmitters AB in ME/CFS

Based on recent studies employing the monoclonal anti-CD20 antibody rituximab [9] in patients with ME/CFS there is reason to believe that the reduction of these neurotransmitters AB may be a promising target for therapy. In these studies, depletion of CD20+ B cells with rituximab led in a majority of patients with ME/CFS to a complete or at least partial remission. Since this beneficial effect occurred only several months after initiation of treatment with rituximab, it may be attributed to the wash-out of the autoantibodies due to the elimination of the short-lived antibody producing plasma cells related to the CD20+ memory B cells. A more immediate therapeutic strategy with less side effects may involve immune apheresis or other forms of extracorporeal apheresis such a neuro- or cerebropheresis [10].

Using immunoadsorption in patients with post-infectious ME/CFS with an IgG-binding column for 5 days in an observational study with ten patients induced a rapid improvement in seven patients [11, 12]. We have also used extracorporeal apheresis (INUSpheresis) enabling a significant reduction of total IgG and all neurotransmitter receptors of up to 50% as well as inflammatory proteins such as CRP or RANTES. In a clinical observation in patients with ME/CFS, extracorporeal apheresis used over either 2 or 4 days induced a significant improvement of symptoms based on disease-specific scores. ME/CFS patients with a clear-cut infection-triggered increase of autoantibodies seemed to have the most significant and sustainable clinical benefits. However, it is also highly conceivable that clinical improvement seen in patients subjected to therapeutic extracorporeal apheresis may have multiple causes, not only related to reduction in autoantibodies against neurotransmitters but also other yet unknown autoantibodies, inflammatory proteins, and rheological factors.

## ME/CFS in post-COVID-19 patients and therapeutic options

Post-COVID-19 syndrome is frequently associated with continuing respiratory problems and debilitating fatigue. There is now increasing evidence that a great variety of autoantibodies may be driving severe forms of COVID-19. These autoantibodies may also play a crucial role in the extended multi-organ illness persevering for months in patients with “long-COVID-19” [13]. Furthermore, orthostatic cerebral hypoperfusion, hypotension, and small fiber neuropathy have been described [14]. The symptoms that occur in a large number of patients following severe COVID-19 disease but even in many cases following mild SARS-CoV-2 infection are similar to the clinical symptoms of other forms of infection-triggered ME/CFS. Although, oxidative stress may contribute to this syndrome replacement [15] of antioxidants and vitamins according to our experience are insufficient to allow for any major improvement of the clinical signs. Similarly, cognitive behavioral therapies are not likely to be sufficient to help in this situation [16]. Hypothalamic–pituitary–adrenal–hypofunction has been described in patients with ME/CFS as a consequence of activated immune-inflammatory pathways [17].

Most patients with severe Covid-19 disease have been receiving dexamethasone and may have an adrenal pre-damaged by the inflammatory process [18]. Therefore, there may be a predisposition for adrenal insufficiency [19] explaining some of the symptoms of the ME/CFS in post-COVID-19 patients. However, we have tested HPA-axis function in patients post-COVID-19 that have received dexamethasone and did not observe any blunting of adrenal cortisol levels (unpublished observation). Based on these findings, replacement with hydrocortisone may not be indicated to improve the severe signs of patients with a post-COVID-19 fatigue syndrome. First, results in our clinic, however, demonstrate elevations of neurotransmitter receptor antibodies similar to what we have seen in other forms of infection-triggered ME/CFS. If these findings can be confirmed in larger number of patients extracorporeal apheresis or immunadsorption may offer a simple and effective treatment option to relieve many of the cumbersome symptoms of these patients. If SARS-CoV-2 can trigger autoimmune disease [20], inducing a large variety of autoantibodies damaging pulmonary cardiovascular and/or nervous system-specific immunosuppressive treatments may be a treatment option. However, a long-term medical immunosuppression or complete removal of immunoglobulins by immune adsorption may also remove protective anti-SARS-CoV-2 antibodies. Therefore, a strategy of extracorporal apheresis allowing a significant reduction of damaging autoantibodies while maintaining an adequate immune defense would be more appropriate. The first evidence gained from investigation of three post-COVID-19 patients in our own centrum showed that extracorporeal apheresis (INUSpherese) could significantly reduce levels of neurotransmitter autoantibodies and alleviate symptoms of CFS, while maintaining sufficient the patient’s anti-SARS-CoV-2 antibodies. The latter observation, however, may not substantially indicate that INUSpherese show certain IgG specificity, but instead can reflect differential kinetics of AB production. We propose using an App providing the criteria for ME/CFS for recruitment, registration, and monitoring of patients and performance of future clinical trials, which method in our opinion may rise more awareness of this clinical condition among younger patients.
